# Left atrial anatomy and atrial arrhythmia recurrence after pulsed-field ablation

**DOI:** 10.1007/s10840-026-02435-2

**Published:** 2026-09-16

**Authors:** Mikko Mutti, David Spreen, Jonas Brügger, Fabian Jordan, Nicolas Schaerli, Albin Bislimaj, Behnam Subin, Felix Mahfoud, Christian Sticherling, Michael Kühne, Patrick Badertscher, Sven Knecht, Philipp Krisai

**Affiliations:** 1 Department of Cardiology, University Hospital Basel, University of Basel, Basel, Switzerland; 2 Cardiovascular Research Institute Basel, University Hospital Basel, University of Basel, Basel, Switzerland

**Keywords:** Atrial fibrillation, Pulsed field ablation, Pulmonary vein isolation, Left atrial anatomy, Pre-interventional imaging

## Abstract

**Background:**

The impact of left atrial (LA) anatomical features on atrial arrhythmia (AA) recurrence after pulsed-field (PF)-based pulmonary vein isolation (PVI) is unknown. This study aimed to assess LA anatomical features associated with long-term AA recurrence after PVI.

**Methods:**

We prospectively enrolled patients undergoing PVI with a pentaspline PF-catheter at a tertiary center from June 2023 to September 2024. AA recurrence was assessed at 3, 6 and 12 months with Holter-ECGs and symptom-driven monitoring. The following anatomical features were assessed in pre-interventional CT or MR imaging: pulmonary vein (PV) ostial diameters, circularity, cross-sectional area, and inter-carina distances. Regression models adjusted for established risk factors were used for their associations with AA recurrence.

**Results:**

Among 287 patients (median age 66 years; 34% female; 61% paroxysmal AF), 98 (34%) experienced recurrence at a median of 6 months after PVI. In adjusted regression analysis, a larger minimal diameter of the right superior PV (OR 1.11 per 1 mm increase, 95% CI (1.02–1.21); *p* = 0.021), and higher circularity indices of the left inferior PV (OR 1.04 per 0.01 increase, 95% CI (1.01–1.07); *p* = 0.005) and the right superior PV (OR 1.03 per 0.01 increase, 95% CI (1.00–1.06); *p* = 0.040) were associated with AA recurrence. There was no interaction for catheter size but some effect modification for the number of PF-applications.

**Conclusion:**

Specific anatomical features of the right superior and left inferior PV were associated with recurrence after PF-based PVI. Although PF-based PVI appears suitable across diverse LA anatomies, the prognostic value of these anatomical characteristics warrants further investigation.

**Graphical Abstract:**

**Figure:**
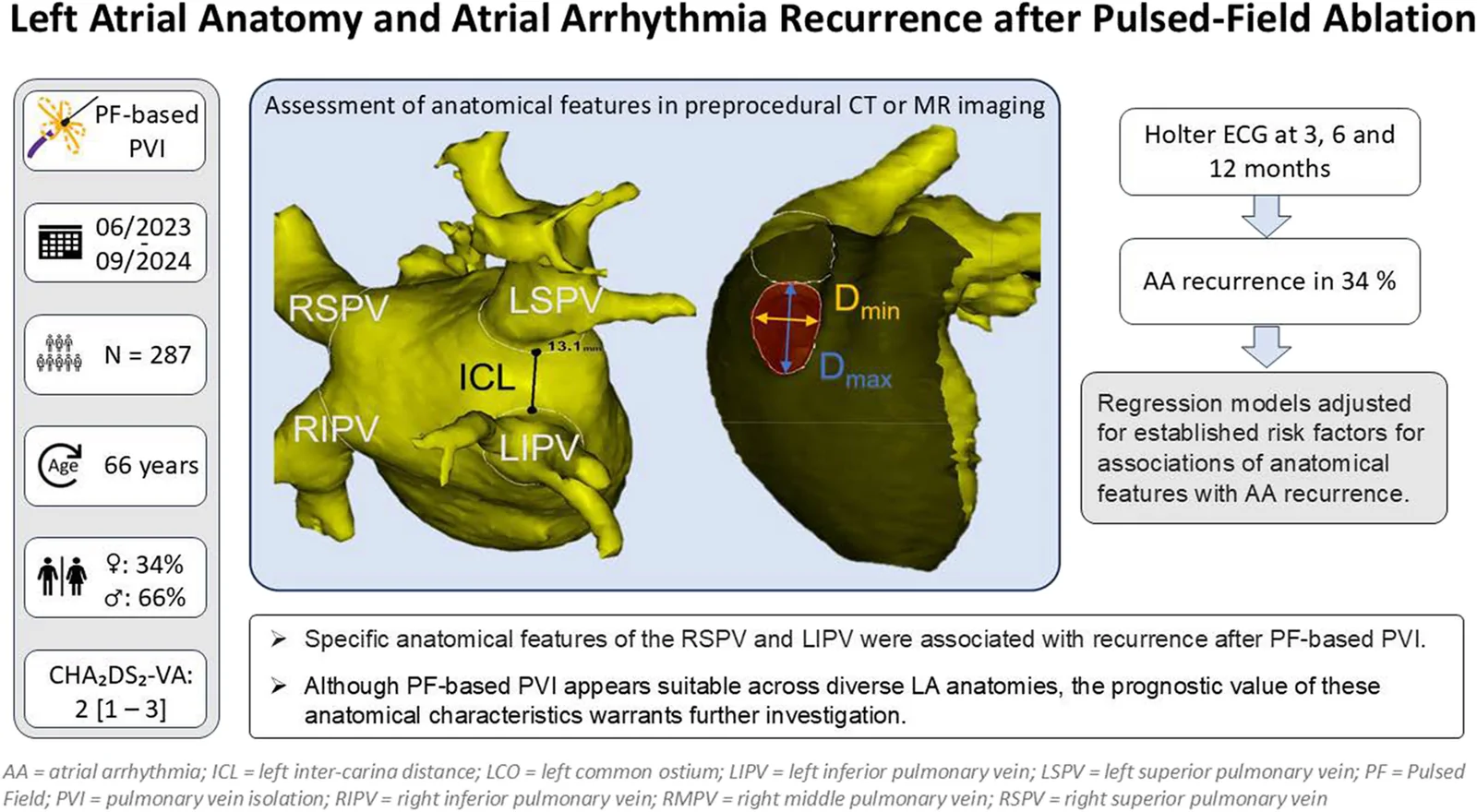

**Supplementary Information:**

The online version contains supplementary material available at https://doi.org/10.1007/s10840-026-02435-2.

## Introduction

Pulsed-field ablation (PFA) has emerged as a safe and efficient ablation technology for pulmonary vein (PV) isolation (PVI) in atrial fibrillation (AF). While PFA-based PVI is associated with shorter procedure times and a potentially more favorable safety profile compared with thermal ablation, atrial arrhythmia (AA) recurrence rates are similar between the two technologies [1–4]. Mandatory remapping studies have demonstrated durable PV isolation after PFA-based PVI in up to 96% of PVs [5], with high durability observed consistently across PFA catheter designs [6]. However, at least one reconnected PV is found in 55–79% of patients undergoing clinically indicated redo procedures for AA recurrence [7–9]. This variation likely reflects operator experience, with PVI durability improving beyond the initial learning curve [10]. Reconnections predominantly occurred in right-sided and superior PVs, suggesting that anatomical features may influence lesion durability and long-term rhythm outcomes after PFA-based PVI [5, 9].

Left atrial (LA) and PV anatomy are independent predictors of AA recurrence following PVI using cryoballoon (CBA) or radiofrequency ablation (RFA) [11–15]. While focal PFA-systems are expected to adapt to anatomical characteristics similar to focal RFA catheters, single-shot PFA systems might depend more on LA anatomy [16]. In redo procedures due to AA recurrences after PVI with the pentaspline PFA-system, PV diameter and angulation were associated with reconnected PVs [17]. However, PV reconnection is a vein-specific finding assessed only in the subset of patients undergoing redo procedures, and is not synonymous with AA recurrence at the patient level. Little is known about the association between LA anatomy and AA recurrence after PVI with the pentaspline PFA-system.

In this study, we investigated the associations between LA anatomical characteristics derived from pre-interventional imaging and AA recurrence after PVI performed with the pentaspline PFA-system.

## Methods

### Study design and participants

The current study is based on the prospective SWISS-AF-PVI registry (NCT03718364) at the University Hospital Basel, Switzerland. For the present study we included a specific cohort consisting of all consecutive patients from the registry with paroxysmal or persistent AF who underwent their first PVI with the pentaspline PFA-system between June 2023 and September 2024. This timeframe was selected to allow a clinical follow-up of 365 days for all included patients. Exclusion criteria were prior PVI or additional PFA applications outside the PVs. All participants were at least 18 years old and provided written informed consent. The study was conducted in accordance with the principles of the Declaration of Helsinki and was approved by the local ethics committees.

### Procedures

#### Preprocedural imaging

Preprocedural LA imaging was performed using either computed tomography (CT) or magnetic resonance imaging (MRI).

MRI was performed on a 1.5-Tesla scanner (Magnetom Avanto/Espree, Siemens, Germany) using phased array body coils. A respiratory- and ECG-gated three-dimensional balanced steady-state free precession sequence was acquired in axial orientation, covering the entire left atrium, both before and after contrast injection (0.1 mmol/kg at 2 ml/s, MultiHance, Bracco, Milano, Italy), with the following parameters: TR/TE = 3.4/1.5 ms; flip angle 90°; slice thickness 1.2 × 1.5 mm^3^.

Preprocedural cardiac CT scans were performed on a Siemens SOMATOM Definition Flash scanner during inspiratory breath-hold with ECG-triggering. Contrast medium (90 ml Ultravist 370, Bayer Healthcare) was injected via the left brachial vein at 4.5 ml/s, followed by a 20 ml saline chaser; the procedure was repeated after 3 min. Bolus tracking was triggered in the ascending aorta, and images were reconstructed in 0.75-mm axial slices.

In addition, all patients underwent transoesophageal echocardiography to exclude LA thrombus and for echocardiographic LA measurements the day before the procedure.

#### Measurement of anatomical parameters

Anatomical structures were segmented and reconstructed in CARTO 3 (CartoSeg, Biosense Webster, Diamond Bar, CA, USA). After visualizing the endocardial surface of the LA, the PV ostia were defined anatomically at the inflection points where the walls of the LA and PVs visually intersect, as described by Kato et al. [18]. While independent validation data for these proprietary segmentation tools are limited [19, 20], the integration of pre-procedural CT or MR imaging into 3D electroanatomic mapping systems is an established approach for anatomical reconstruction of the LA and PVs [21]. These anatomical landmarks served as reference points for manually delineating the PV ostia. The maximum (D_max_) and minimum (D_min_) diameters of the PVs were measured on the previously defined ostia perpendicular to each other. PV circularity index was calculated by dividing the minimum by the maximum PV diameter (measured perpendicularly), with values closer to 1 indicating a more circular ostium. Cross-sectional area (CSA) was calculated using the formula $\:CSA=\pi\:\:\times\:0.25\:\times\:{D}_{min}\times\:{D}_{max}$.

**Fig. 1:**
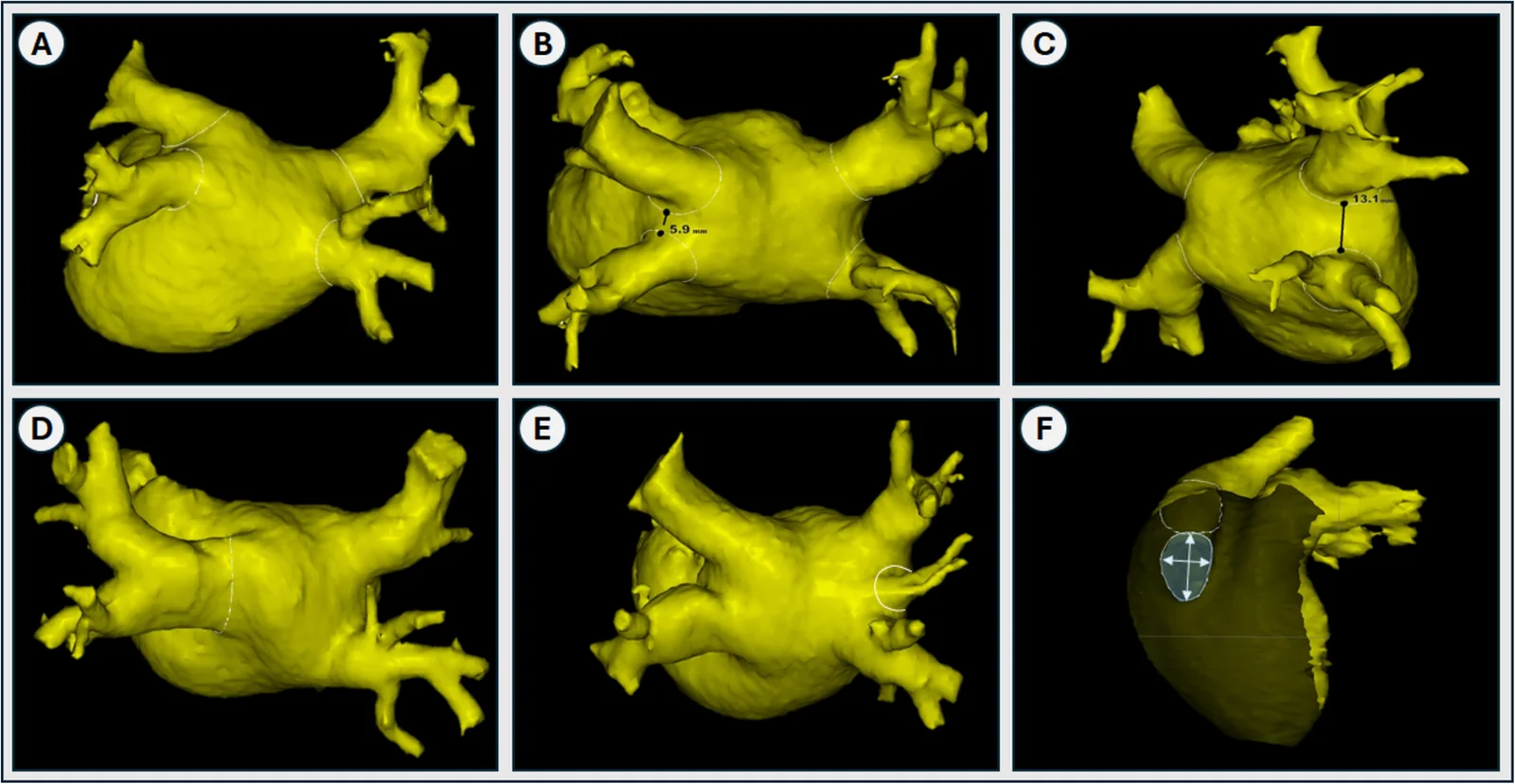
Visualization matrix of measured anatomical features. Panel **A**: CARTO-segmented left atrium in posteroanterior view; Panel**B**: left inter-carina distance; Panel **C**: right inter-carina distance; Panel **D**: left common ostium; Panel **E**: right middle pulmonary vein; Panel **F**: pulmonary vein ostial dimensions (D_min_ and D_max_)

The left and right inter-carina distances, defined as the shortest distance between the ipsilateral PV ostia, were measured using an electronic calliper. A left common ostium (LCO) was defined when the distance along the anterior ridge from the LA ostium to the PV bifurcation had a minimum length of 10 mm. The presence of a right middle pulmonary vein (RMPV) was defined as an independent atrio-pulmonary venous junction located between the RSPV and RIPV and was assessed visually. Exemplary measurements are shown in Fig. 1.

LA segmentation and anatomical measurements were performed by three trained investigators. Any inconsistencies between the observers were resolved by consensus. To assess inter-observer variability, a subset of ten cases was remeasured in a blinded manner by the initial observer and independently by a second observer.

#### Pulmonary vein isolation

Sedation was induced using a combination of midazolam, fentanyl, and propofol. Femoral vascular access was established under ultrasound guidance in all cases. Transseptal puncture was performed under fluoroscopic guidance. Anticoagulation was achieved through intravenous administration of heparin to maintain an activated clotting time of 350 s.

PVI with the pentaspline PFA-system (Farawave, Boston Scientific, Natick, MA, USA) was performed as previously described [22]. Briefly, following transseptal puncture, a J-tip guidewire (Amplatz Extra-Stiff, 0.035”) was used to cannulate the PVs while the PFA catheter was positioned within the LA. Contact guidance was based on fluoroscopy and visual assessment of intracardiac electrogram morphology. Four applications with a voltage of 2 kV in both basket and flower configurations with 30–40° rotation after two applications were applied per vein as recommended by the manufacturer. Additional PV ablation applications were at the operator’s discretion. A subset of patients underwent a simplified protocol with two applications in each configuration per vein, with rotation after the first application [23]. In left common PV, ablations were performed with the guidewire positioned sequentially in the superior and inferior branches while maintaining the catheter at the LCO with similar number of applications. The choice between the 31–35 mm pentaspline catheter was at the operator’s discretion. Acute PV isolation was assessed based on local bipolar electrogram reduction and testing for exit block, as appropriate. In line with the SINGLE SHOT CHAMPION study, no routine post-ablation electroanatomical remapping was performed, as acute pulmonary vein isolation was shown to be adequately assessed based on exit block alone [24].

### Follow-up and endpoint definition

The follow-up protocol consisted of outpatient visits at 3, 6, and 12 months, including physical examination, 12-lead ECG, and 24-hour to 7-day Holter ECG monitoring. In patients with implantable cardiac devices (*n* = 39, 13.6%), rhythm monitoring was performed using device data. If symptomatic arrhythmia occurred outside the scheduled follow-ups, the event was documented using a 12-lead ECG, Holter ECG, or another approved method of heart rhythm recording in accordance with the ESC 2024 guidelines [25]. Anti-arrhythmic drug therapy was discontinued directly after the procedure or at the 3 month follow-up visit the latest.

To evaluate the impact of LA anatomical features on long-term procedural efficacy, the primary endpoint was defined as AA recurrence after a blanking period of 60 days up to 1 year of follow-up. AA included AF, atrial tachycardia and non-CTI dependent atrial flutter lasting at least 30 s. AA during the blanking period were classified as recurrences if they persisted beyond the blanking period or led to a redo procedure.

### Statistical analysis

Baseline characteristics are shown overall and stratified by presence or absence of AA recurrence. The distribution of continuous variables was assessed using the Shapiro-Wilk test. Continuous variables are presented as mean (standard deviation (SD)) or median (interquartile range (IQR)), depending on the distribution, and compared using unpaired Student t tests or the Mann-Whitney U tests, as appropriate. Categorical variables are shown as counts (percentages) and compared using χ^2^ test or Fisher exact test, as appropriate. Because the primary endpoint was defined as the occurrence of AA recurrence during follow-up, recurrence was analysed as a binary outcome and univariate logistic regression models were used to evaluate associations with individual anatomical features. Models were further adjusted for established AA recurrence risk factors including age, sex, body mass index, indexed LA volume and type of AF. For sensitivity analysis, imaging modality was added as a covariate to the adjusted model. We additionally performed interaction analyses for catheter size (31 mm vs. 35 mm) and number of applications (≥ 32 and < 32). The cut-off of 32 applications was chosen to separate patients who received at least the full standard protocol (≥ 32), as recommended by the manufacturer, from those treated with a reduced, non-standard protocol (< 32). As this analysis was exploratory, we did not correct for multiple testing and interpreted p-values as continuous values. All statistical analyses were performed using R version 4.4.2 (The R Foundation for Statistical Computing, Vienna, Austria) and RStudio 2024.12.1 + 563 (Posit Software PBC, Boston, MA, USA). ChatGPT (OpenAI) was used to assist with debugging and optimization of R scripts for statistical analysis in RStudio. The authors reviewed and edited all outputs and take full responsibility for the analyses.

## Results

### Study population and AF ablation procedures

Baseline characteristics of the 287 included patients are shown in Table 1. The median age was 66 [58–73] years and 97 patients (34%) were women. The median CHA₂DS₂-VA Score was 2 [1–3] and 174 patients (61%) had paroxysmal AF. Median LA volume index (LAVI) and left ventricular ejection fraction (LVEF) were 36 [29–44] ml/m^2^ and 58 [53–63] %, respectively. Mean LA diameter was 40.1 ± 7.1 mm. Compared to patients without AA recurrence, patients with recurrence were older (67 vs. 64 years), more frequently female (43% vs. 29%), had more often non-paroxysmal AF (51% vs. 33%) and AF at the time of PVI (45% vs. 27%). LA diameter (42.7 vs. 38.8 mm) and LAVI (42.0 vs. 35.3 ml/m^2^) were also larger in patients with AA recurrence.

**Table 1:** Baseline characteristics overall and stratified by recurrence status

	Overall (*N* = 287)	No recurrence (*N* = 189)	Recurrence (*N* = 98)	*p*-value
Age (years)	66 [58–73]	65 [58–72]	68 [61–73]	0.038
Sex (female)	97 (33.8%)	55 (29.1%)	42 (42.9%)	0.027
BMI [kg/m²]	27 [24–29]	26 [24–29]	27 [24–31]	0.112
Paroxysmal AF	174 (60.6%)	126 (66.7%)	48 (49.0%)	0.005
Rhythm before PVI				0.008
* SR*	190 (66.2%)	137 (72.5%)	53 (54.1%)	
* AF*	95 (33.1%)	51 (27.0%)	44 (44.9%)	
* other*	2 (0.7%)	1 (0.5%)	1 (1.0%)	
CHA₂DS₂-VA Score	2 [1–3]	2 [1–3]	2 [1–3]	0.178
LVEF [%]	58 [53–63]	59 [54–63]	58 [50–63]	0.171
LA diameter [mm]	40.10 ± 7.1	38.75 ± 6.8	42.71 ± 6.9	< 0.001
LAVI [ml/m^2^]	36 [29–44]	35 [27–41]	40 [34–48]	< 0.001
Medical history				
Hypertension	158 (55.1%)	103 (54.5%)	55 (56.1%)	0.891
Diabetes	31 (10.8%)	19 (10.1%)	12 (12.2%)	0.714
Hypercholesterolemia	111 (38.7%)	68 (35.9%)	43 (43.9%)	0.240
Coronary artery disease	33 (11.5%)	19 (10.1%)	14 (14.3%)	0.384
Prior myocardial infarction	16 (5.6%)	9 (4.8%)	7 (7.1%)	0.574
Active smoking	23 (8.0%)	19 (10.1%)	4 (4.1%)	0.124
Prior stroke/TIA	16 (5.6%)	14 (7.4%)	2 (1.0%)	0.093

Values are presented as mean (standard deviation), median (IQR) or count (percentage). Abbreviations: *AF* atrial fibrillation, *BMI* body mass index, *LA* left atrial, *LAVI* left atrial volume index, *LVEF* left ventricular ejection fraction, *PVI* pulmonary vein isolation, *SR* sinus rhythm, *TIA* transient ischemic attack

Detailed procedural characteristics are shown in Table 2. The mean total procedural duration was 44.8 ± 17.5 min and the mean LA dwell time 16.9 ± 7.3 min. The mean total number of PFA applications during the procedure was 32.5 ± 10.4 and acute PVI was successfully achieved in 100% of patients. The 31 mm pentaspline catheter was used in 82% of cases and the 35 mm catheter in the remaining. Procedural characteristics were similar between patients with and without AA recurrence.

**Table 2:** Procedural characteristics overall and stratified by recurrence status

	Overall (*N* = 287)	No recurrence (*N* = 189)	Recurrence (*N* = 98)	*P* value
Total procedural duration [min]	44.81 ± 17.5	44.57 ± 18.0	45.29 ± 16.5	0.541
LA dwell time [min]	16.91 ± 7.3	17.31 ± 7.9	16.14 ± 6.0	0.456
Fluoroscopy time [min]	10.21 ± 4.5	10.10 ± 4.6	10.44 ± 4.3	0.388
Fluoroscopy dose [Gycm^2^]	568.16 ± 689.0	558.79 ± 728.9	586.14 ± 608.2	0.168
Total applications	32.52 ± 10.4	32.66 ± 10.7	32.26 ± 9.8	0.668
Catheter size				0.405
* 31 mm*	234 (81.5%)	151 (79.9%)	83 (84.7%)	
* 35 mm*	53 (18.5%)	38 (20.1%)	15 (15.3%)	

Values are presented as mean (standard deviation) or count (percentage). Abbreviations: *Gycm*^*2*^ gray per cm^2^, *LA* left atrial

### Left atrial anatomical characteristics

LA anatomical characteristics overall and stratified by AA recurrence are shown in Table 3. Preprocedural imaging was performed with MRI in 153 patients (53%) and with CT in 134 patients (47%). A LCO and RMPV were present in 63 (22%) and 39 (14%) patients, respectively. One patient had a right common ostium. Compared to patients without AA recurrence, patients with recurrence tended to have larger minimal and maximal PV diameters and a larger cross-sectional PV area, except for the LIPV. The circularity index had similar measurements for the LSPV and RIPV but was larger for the RSPV and LIPV in patients with recurrence compared to those without recurrence. Analysis of inter-observer reproducibility showed a mean correlation coefficient of 0.95, indicating consistent and reproducible measurements.

**Table 3:** Left atrial anatomical characteristics overall and stratified by recurrence status

	Overall (*N* = 287)	No recurrence (*N* = 189)	Recurrence (*N* = 98)	*p*-value
Preprocedural Imaging				0.053
CT	134 (46.7%)	80 (42.3%)	54 (55.1%)	
MRI	153 (53.3%)	109 (57.7%)	44 (44.9%)	
RMPV	39 (13.6%)	28 (14.8%)	11 (11.2%)	0.509
LCO	63 (21.9%)	38 (20.1%)	25 (25.5%)	0.314
Inter-Carina Distance Left [mm]	3.9 [2.7–5.7]	3.9 [2.8–5.6]	4.1 [2.7–6.2]	0.628
Inter-Carina Distance Right [mm]	6.1 [4.0–9.9]	6.1 [3.9–10]	6.3 [4.2–9.8]	0.893
LSPV (*n* = 224)				
Max. Diameter [mm]	22 [20–25]	22 [20–25]	23 [21–26]	0.030
Min. Diameter [mm]	15.79 ± 3.4	15.58 ± 3.3	16.25 ± 3.4	0.212
Circularity Index	0.7 [0.6–0.8]	0.7 [0.6–0.8]	0.7 [0.6–0.8]	0.633
CSA [cm^2^]	3.0 [2.3–4.1]	2.9 [2.3–3.8]	3.4 [2.5–4.7]	0.015
LIPV (*n* = 224)				
Max. Diameter [mm]	19.38 ± 2.9	19.41 ± 3.1	19.31 ± 2.4	0.968
Min. Diameter [mm]	14.06 ± 3.3	13.77 ± 3.4	14.66 ± 3.1	0.068
Circularity Index	0.73 ± 0.1	0.71 ± 0.1	0.76 ± 0.1	0.010
CSA [cm^2^]	2.1 [1.6–2.6]	2.1 [1.6–2.6]	2.2 [1.7–2.8]	0.304
LCO (*n* = 63)				
Max. Diameter [mm]	33.12 ± 6.0	31.67 ± 5.0	35.26 ± 6.7	0.054
Min. Diameter [mm]	22 [19–26]	20 [18–23]	25 [19–27]	0.040
Circularity Index	68 [60–79]	66 [58–80]	69 [64–79]	0.430
CSA [cm^2^]	5.5 [4.3–7.6]	4.8 [4.0–6.2]	7.1 [4.7–7.9]	0.016
RSPV (*n* = 286)				
Max. Diameter [mm]	22.89 ± 3.6	22.66 ± 3.5	23.35 ± 3.7	0.139
Min. Diameter [mm]	18.75 ± 3.3	18.24 ± 3.1	19.74 ± 3.5	< 0.001
Circularity Index	0.8 [0.8–0.9]	0.8 [0.7–0.9]	0.9 [0.8–0.9]	0.005
CSA [cm^2^]	3.3 [2.7 −4.0]	3.2 [2.7–3.8]	3.6 [2.9–4.2]	0.006
RIPV (*n* = 286)				
Max. Diameter [mm]	20.58 ± 3.5	20.28 ± 3.3	21.14 ± 3.8	0.062
Min. Diameter [mm]	17.33 ± 3.3	17.01 ± 3.0	17.93 ± 3.7	0.025
Circularity Index	0.8 [0.8–0.9]	0.8 [0.8–0.9]	0.8 [0.8–0.9]	0.365
CSA [cm^2^]	2.8 [2.2–3.4]	2.7 [2.2–3.2]	2.9 [2.3–3.7]	0.023

Values are presented as mean (standard deviation), median (IQR) or count (percentage). Abbreviations: *CSA* cross-sectional area, *CT* computed tomography, *LCO* left common ostium, *LIPV* left inferior pulmonary vein, *LSPV* left superior pulmonary vein, *MRI* magnetic resonance imaging, *RIPV* right inferior pulmonary vein, *RMVP* right middle pulmonary vein, *RSPV* right superior pulmonary vein

### Association of atrial arrhythmia recurrence and anatomical characteristics

During follow-up, 98 patients (34%) experienced an AA recurrence, at a median of 6 (IQR 3–10) months after the index procedure. AA were AF in 86, atrial tachycardia in 9 and non-CTI dependent atrial flutter in 3 patients. For patients without recurrence, median follow-up was 12 (IQR 8–12) months.

**Fig. 2:**
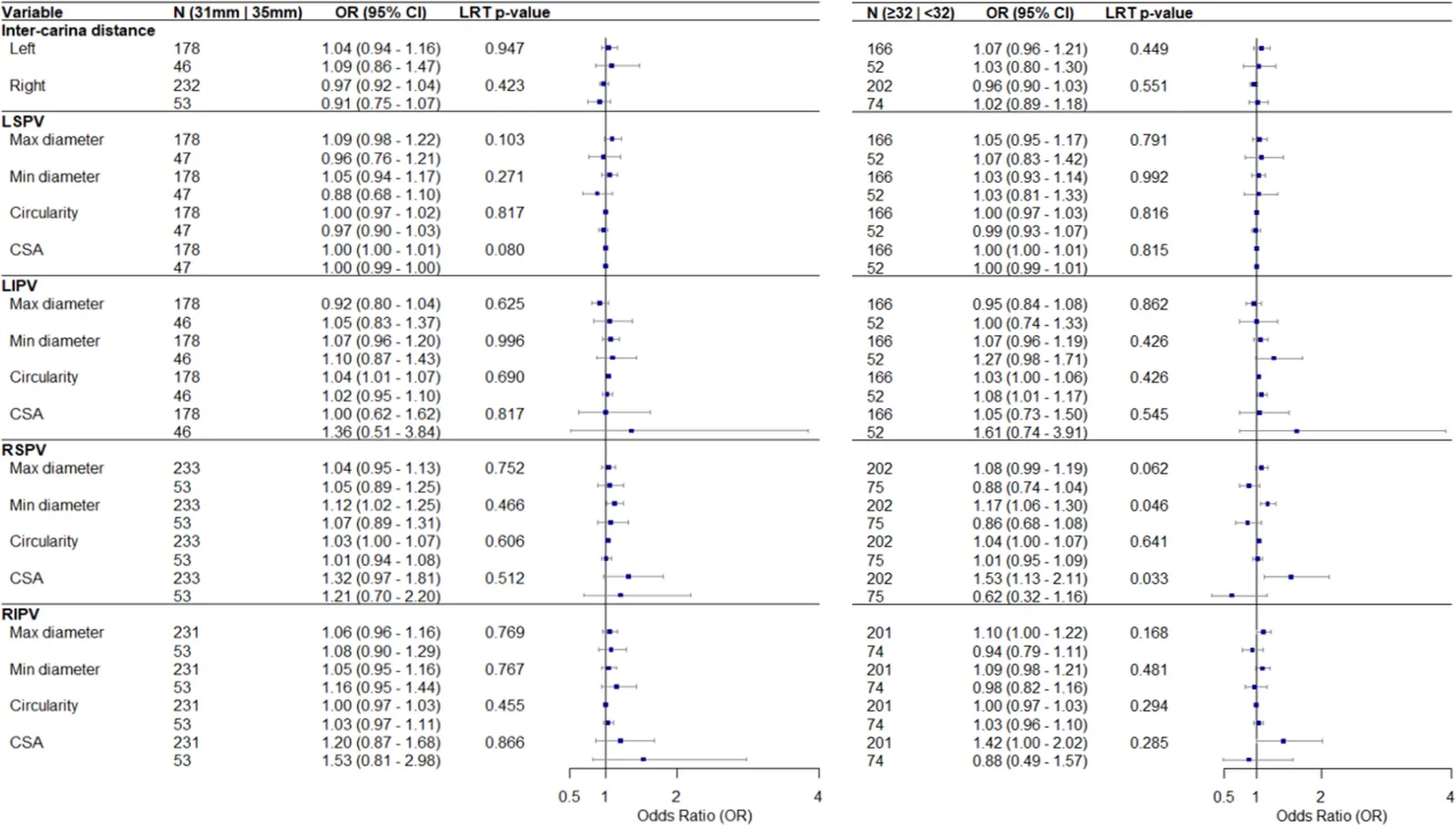
Interaction between device size (31 mm vs. 35 mm; left panel) or ablation protocol (≥32 or <32 applications; right panel) and anatomical predictors of atrial arrhythmia recurrence. Adjusted for age, sex, BMI, LAVI and type of AF

Results of unadjusted and adjusted regression models for the associations of LA anatomical features and AA recurrence are shown in Table 4. In adjusted models, the minimal diameter (OR 1.11 per 1 mm increase, 95% CI 1.02–1.21; *p* = 0.021) and circularity index (OR 1.03 per 0.01 increase, 95% CI 1.00–1.06; *p* = 0.04) of the RSPV, and the circularity index (OR 1.04 per 0.01 increase, 95% CI 1.01–1.07; *p* = 0.005) of the LIPV were associated with higher odds for AA recurrence. No other investigated LA anatomical feature including inter-carina distances or the presence of an anatomical variant PV was associated with AA recurrence. Results were consistent for the 31 vs. 35 mm ablation catheters (all p-values for interaction > 0.05). We found an effect modification for the number of applications of the RSPV: larger minimal diameter and CSA were associated with a higher AA recurrence rate in patients with ≥ 32 PFA applications (Fig. 2).

**Table 4:** Association of left atrial anatomical characteristics with atrial arrhythmia recurrence

	Unadjusted Model			Adjusted Model		
	Odds Ratio	95% CI	*p*-value	Odds Ratio	95% CI	*p*-value
RMPV (*n* = 39) (presence vs. absence)	0.727	0.332–1.494	0.401	0.555	0.231–1.247	0.168
LCO (*n* = 63) (presence vs. absence)	1.407	0.783–2.502	0.248	1.506	0.796–2.828	0.204
Inter-Carina Distance Left (*n* = 224) (per 1 mm increase)	1.052	0.964–1.149	0.252	1.051	0.955–1.163	0.311
Inter-Carina Distance Right (*n* = 286) (per 1 mm increase)	1.000	0.950–1.051	0.996	0.970	0.915–1.027	0.306
LSPV (*n* = 224)						
Max. Diameter (per 1 mm increase)	1.085	1.000–1.179	0.052	1.051	0.957–1.156	0.299
Min. Diameter (per 1 mm increase)	1.060	0.976–1.152	0.167	1.023	0.931–1.124	0.638
Circularity Index (per 0.01 increase)	1.159	0.143–9.397	0.890	0.831	0.080–8.556	0.876
CSA (per 1 cm^2^ increase)	1.323	0.981–1.792	0.067	1.144	0.811–1.613	0.438
LIPV (*n* = 224)						
Max. Diameter (per 1 mm increase)	0.989	0.896–1.091	0.821	0.950	0.846–1.063	0.372
Min. Diameter (per 1 mm increase)	1.087	0.997–1.187	0.060	1.082	0.983–1.194	0.114
Circularity Index (per 0.01 increase)	1.476	1.103–2.005	0.010	1.039	1.012–1.067	0.005
CSA (per 1 cm^2^ increase)	1.215	0.839–1.761	0.302	1.097	0.721–1.671	0.666
LCO (*n* = 63)						
Max. Diameter (per 1 mm increase)	1.115	1.018–1.238	0.027	1.106	0.984–1.262	0.106
Min. Diameter (per 1 mm increase)	1.080	0.995–1.183	0.076	1.045	0.946–1.162	0.399
Circularity Index (per 0.01 increase)	1.157	0.692–1.963	0.578	0.975	0.011–86.78	0.991
CSA (per 1 cm^2^ increase)	1.244	1.020–1.565	0.042	1.161	0.905–1.528	0.259
RSPV (*n* = 286)						
Max. Diameter (per 1 mm increase)	1.056	0.986–1.133	0.122	1.030	0.953–1.114	0.455
Min. Diameter (per 1 mm increase)	1.154	1.068–1.253	< 0.001	1.109	1.017–1.214	0.021
Circularity Index (per 0.01 increase)	1.041	1.014–1.070	0.003	1.031	1.002–1.062	0.040
CSA (per 1 cm^2^ increase)	1.427	1.130–1.819	0.003	1.269	0.976–1.662	0.078
RIPV (*n* = 286)						
Max. Diameter (per 1 mm increase)	1.072	1.000–1.152	0.053	1.056	0.973–1.148	0.192
Min. Diameter (per 1 mm increase)	1.091	1.011–1.179	0.026	1.064	0.976–1.161	0.162
Circularity Index (per 0.01 increase)	1.008	0.982–1.036	0.544	1.008	0.983–1.037	0.542
CSA (per 1 cm^2^ increase)	1.356	1.058–1.750	0.017	1.257	0.941–1.686	0.122

Odds ratios in adjusted models are adjusted for age, sex, BMI, LAVI and type of AF. Abbreviations: *CSA* cross-sectional area, *LCO* left common ostium, *LIPV* left inferior pulmonary vein, *LSPV* left superior pulmonary vein, *RIPV* right inferior pulmonary vein, *RMVP* right middle pulmonary vein, *RSPV* right superior pulmonary vein

## Discussion

To the best of our knowledge, this is the first study to investigate the association between LA anatomical features and AA recurrence after PVI with the pentaspline PFA-system. Patients with AA recurrence tended to exhibit larger PV diameters, cross-sectional areas and circularity indices compared to patients without AA recurrence. These anatomical characteristics of the RSPV and LIPV, but not of the remaining PVs, were associated with AA recurrence after adjustment for confounders. Our findings suggest that it may be worthwhile to investigate in future studies whether integrating anatomical characteristics into single-shot PFA-based PVI strategies influences ablation outcomes.

Larger ostial diameters, cross-sectional areas and circularity indices of some PVs - indicating a more circular and enlarged PV ostium - were independently associated with AA recurrence after PFA-based PVI. Anatomical PV variants including LCO and RMPV, were not associated with AA recurrence in our cohort [16]. By contrast, in CBA, larger ostial diameters and increased PV ovality, indicating similarly enlarged but more oval PVs, have been linked to increased procedural difficulty and PV reconnections [14, 26]. Moreover, larger PV cross-sectional areas, RSPV diameters and the presence of a LCO and/or RMPV were independent predictors of AF recurrence after CBA [11, 12, 27]. In point-by-point RF ablation, RSPV orientation predicted AF-free survival, whereas PV anatomical variations and ostial parameters, including cross-sectional area, effective diameter, and eccentricity (defined identically to our circularity index), showed no association [15]. Concerning anatomical characteristics after PF-based PVI, Simavonian et al. reported in a multicenter cohort that larger long-axis diameters and greater differences in lateral PV angulation were independently associated with PV reconnections, although the effect size was limited [17]. In two other studies with similar patient populations, PV reconnections were predominantly localized to the right-sided and superior PVs [5, 9]. Whether this is due to procedural factors, such as transseptal catheter trajectory and catheter apposition, anatomical characteristics, or other unmeasured factors is unclear. After adjustment for potential confounders, we observed moderate but overall inconsistent associations of anatomical characteristics of the RSPV and LIPV with AA recurrence in the present study.

The clinical implications of our current and prior findings regarding the role of PV anatomy in PFA-based PVI remain limited. In sub-analyses, we observed no significant interactions related to catheter size but some effect modification according to the number of applications. To further define the role of LA anatomical features in PFA-based PVI, ideally, prospective trials randomizing operators to availability versus non-availability of preprocedural imaging would be desirable.

### Strengths and limitations

The strengths of this study include the large and well-defined population with consistent and reproducible anatomical measurements. Several limitations should be acknowledged. First, this was a non-randomized observational study, limiting causal inference and rendering the findings susceptible to confounding. Operators were not blinded to LA anatomy and may have selected the catheter size and/or adapted their number of applications based on perceived anatomical complexity, thereby introducing potential performance bias. Second, the sample size limits statistical power and increases the risk of type II error and overestimation of effect sizes, specifically in sub-analyses. Third, we did not perform remapping studies to assess PV reconnection rates and their impact on recurrences, which may be influenced by more than PV anatomy alone. Thus, enlarged or more circular PVs at the time of the first procedure might be a proxy for more advanced forms of AF, that are themselves associated with higher AA-recurrence rates [28]. Fourth, all procedures were performed with a pentaspline PFA catheter, limiting generalizability of our results to other PFA systems. Fifth, contact guidance was based on fluoroscopy and visual assessment of intracardiac electrogram morphology only. We did not use intracardiac echocardiography for direct confirmation of catheter–tissue contact during ablation. Sixth, no routine post-ablation electroanatomical remapping was performed, limiting assessment of acute lesion completeness. Seventh, anatomical measurements were derived from different imaging modalities (CT and MRI), which may have introduced measurement variability. To address this, imaging modality was included as a covariate in a sensitivity analysis (Table S1), with consistent results compared to the main analysis.

## Conclusion

In this prospective cohort of patients undergoing PFA-based PVI, selected LA anatomical characteristics were moderately but inconsistently associated with AA recurrence. Our findings support the effectiveness of the pentaspline PFA-system across different LA anatomies. Further investigation is warranted on whether incorporating anatomical characteristics into PFA-based PVI planning influences AA recurrence rates.

## Supplementary Information

Below is the link to the electronic supplementary material.

Supplementary Material 1

## Data Availability

The data used to support the findings of this study are available from the corresponding author upon request.

## Non-standard abbreviations

CBA: Cryoballoon Ablation
LA: Left Atrium
LAVI: Left Atrial Volume Index
LCO: Left Common Ostium
LIPV: Left Inferior Pulmonary Vein
LSPV: Left Superior Pulmonary Vein
PFA: Pulsed Field Ablation
PV(s): Pulmonary Vein(s)
PVI: Pulmonary Vein Isolation
RIPV: Right Inferior Pulmonary Vein
RMPV: Right Middle Pulmonary Vein
RSPV: Right Superior Pulmonary Vein

## Declarations

### Patient permission

All participants were at least 18 years old and provided written informed consent.
