# Supplementary material for: Left atrial anatomy and atrial arrhythmia recurrence after pulsed-field ablation
Source: J Interv Card Electrophysiol. 2026 Sep 16;69(9):3137–46. doi: 10.1007/s10840-026-02435-2 (PMC13769681; doi:10.1007/s10840-026-02435-2)
Supplement: Supplementary file 1 [file 10840_2026_2435_MOESM1_ESM.docx]

**SUPPLEMENTARY MATERIAL**

**Left Atrial Anatomy and Atrial Arrhythmia Recurrence after Pulsed-Field Ablation**

**Index**

Supplementary Table S1	3

**Supplementary Table S1**: Sensitivity analysis of anatomical predictors of AA recurrence adjusted for imaging modality (CT vs. MRI)

|  | **Adjusted Model 1** | | | **Adjusted Model 2** | | |
| --- | --- | --- | --- | --- | --- | --- |
|  | OR | 95% CI | p-value | OR | 95% CI | p-value |
| **RMPV (n = 39)** (presence vs. absence) | 0.555 | 0.231 - 1.247 | 0.168 | 0.555 | 0.231 - 1.247 | 0.168 |
| **LCO (n = 63)** (presence vs. absence) | 1.506 | 0.796 - 2.828 | 0.204 | 1.506 | 0.796 - 2.828 | 0.204 |
| **Inter-Carina Distance Left (n = 224)** (per 1 mm increase) | 1.051 | 0.955 - 1.163 | 0.311 | 1.043 | 0.945 - 1.157 | 0.412 |
| **Inter-Carina Distance Right (n = 286)** (per 1 mm increase) | 0.970 | 0.915 - 1.027 | 0.306 | 0.972 | 0.925 - 1.020 | 0.295 |
| **LSPV (n = 224)** |  |  |  |  |  |  |
| Max. Diameter (per 1 mm increase) | 1.051 | 0.957 - 1.156 | 0.299 | 1.059 | 0.962 - 1.168 | 0.241 |
| Min. Diameter (per 1 mm increase) | 1.023 | 0.931 - 1.124 | 0.638 | 1.018 | 0.925 - 1.119 | 0.638 |
| Circularity Index (per 0.01 increase) | 0.831 | 0.080 - 8.556 | 0.876 | 0.995 | 0.971 - 1.019 | 0.661 |
| CSA (per 1 cm^2^ increase) | 1.144 | 0.811 - 1.613 | 0.438 | 1.001 | 0.998 - 1.005 | 0.439 |
| **LIPV (n = 224)** |  |  |  |  |  |  |
| Max. Diameter (per 1 mm increase) | 0.950 | 0.846 - 1.063 | 0.372 | 0.952 | 0.848 - 1.067 | 0.398 |
| Min. Diameter (per 1 mm increase) | 1.082 | 0.983 - 1.194 | 0.114 | 1.085 | 0.984 - 1.200 | 0.105 |
| Circularity Index (per 0.01 increase) | 1.039 | 1.012 - 1.067 | 0.005 | 1.039 | 1.012 - 1.068 | 0.005 |
| CSA (per 1 cm^2^ increase) | 1.097 | 0.721 - 1.671 | 0.666 | 1.114 | 0.730 - 1.707 | 0.615 |
| **LCO (n = 63)** |  |  |  |  |  |  |
| Max. Diameter (per 1 mm increase) | 1.106 | 0.984 - 1.262 | 0.106 | 1.106 | 0.984 - 1.261 | 0.106 |
| Min. Diameter (per 1 mm increase) | 1.045 | 0.946 - 1.162 | 0.399 | 1.044 | 0.946 - 1.162 | 0.403 |
| Circularity Index (per 0.01 increase) | 0.975 | 0.011 - 86.78 | 0.991 | 0.999 | 0.955 - 1.045 | 0.952 |
| CSA (per 1 cm^2^ increase) | 1.161 | 0.905 - 1.528 | 0.259 | 1.001 | 0.999 - 1.004 | 0.261 |
| **RSPV (n = 286)** |  |  |  |  |  |  |
| Max. Diameter (per 1 mm increase) | 1.030 | 0.953 - 1.114 | 0.455 | 1.040 | 0.961 - 1.127 | 0.329 |
| Min. Diameter (per 1 mm increase) | 1.109 | 1.017 - 1.214 | 0.021 | 1.124 | 1.029 - 1.233 | 0.011 |
| Circularity Index (per 0.01 increase) | 1.031 | 1.002 - 1.062 | 0.040 | 1.031 | 1.002 - 1.062 | 0.039 |
| CSA (per 1 cm^2^ increase) | 1.269 | 0.976 - 1.662 | 0.078 | 1.327 | 1.014 - 1.755 | 0.042 |
| **RIPV (n = 286)** |  |  |  |  |  |  |
| Max. Diameter (per 1 mm increase) | 1.056 | 0.973 - 1.148 | 0.192 | 1.060 | 0.970 - 1.160 | 0.194 |
| Min. Diameter (per 1 mm increase) | 1.064 | 0.976 - 1.161 | 0.162 | 1.078 | 0.987 - 1.180 | 0.100 |
| Circularity Index (per 0.01 increase) | 1.008 | 0.983 - 1.037 | 0.542 | 1.005 | 0.977 - 1.035 | 0.715 |
| CSA (per 1 cm^2^ increase) | 1.257 | 0.941 - 1.686 | 0.122 | 1.319 | 0.980 - 1.788 | 0.070 |

Odds ratios in adjusted model 1 are adjusted for age, sex, BMI, LAVI and type of AF. Adjusted model 2 is additionally adjusted for imaging modality (CTA or MRI). Abbreviations: CSA = cross-sectional area; LCO = left common ostium; LIPV = left inferior pulmonary vein; LSPV = left superior pulmonary vein; OR = odds ratio; RIPV = right inferior pulmonary vein; RMVP = right middle pulmonary vein; RSPV = right superior pulmonary vein.
